# Effects of congested fixture and matches’ participation on internal and external workload indices in professional soccer players

**DOI:** 10.1038/s41598-022-05792-w

**Published:** 2022-02-03

**Authors:** Gabriel Rodrigues Garcia, Luiz Guilherme C. Gonçalves, Filipe Manuel Clemente, Fábio Yuzo Nakamura, Hadi Nobari, Bruno Luiz Souza Bedo, Angelo Melim Azevedo, Mauro Antonio Guerra, Rodrigo Aquino

**Affiliations:** 1 Research Group in Soccer Science, Department of Sports, Center of Physical Education and Sports, Federal University of Espírito Santo, Vitória, Brazil; 2 Department of Performance Analysis, Botafogo Football Club S/A, Ribeirão Preto, SP Brazil; 3 Department of Science and Technology, Status On Sports (SOSports), Ribeirão Preto, SP Brazil; 4 Escola Superior Desporto e Lazer, Instituto Politécnico de Viana do Castelo, Viana do Castelo, Portugal; 5 Delegação da Covilhã, Instituto de Telecomunicações, Lisboa, Portugal; 6 Associate Graduate Program in Physical Education UPE/UFPB, João Pessoa, PB Brazil; 7 Department of Exercise Physiology, Faculty of Educational Sciences and Psychology, University of Mohaghegh Ardabili, 56199-11367 Ardabil, Iran; 8 Sports Scientist, Sepahan Football Club, Isfahan, Iran; 9 Ribeirão Preto Medical School, University of São Paulo, Ribeirão Preto, Brazil; 10 Department of Physiology, School of Sport Sciences, University of Extremadura, 10003 Cáceres, Spain

**Keywords:** Physiology, Engineering

## Abstract

This study aimed to verify the effects of congested fixture and matches’ participation on internal and external workload indexes in professional Brazilian soccer players. Rate of perceived exertion-based training load (sRPE), distance- and accelerometry-based measures were daily monitored over 119 training sessions and 33 matches performed by 29 male outfield players. Weeks were classified as congested (n = 11, two or more matches within a 7-day period) and regular (n = 10, one match or less within a 7-day period). The players were divided based on the matches’ participation: (1) players who played ≥ 60 min (G1); (2) players who played < 60 min (G2); (3) players who did not participate of the match (G3). The findings showed that independent of the levels of participation during the matches, regular weeks presented greater acute, monotony, and strain indices for internal and external workload measures than congested weeks. The G1 presented the highest values for most of the workload indices in both regular and congested weeks, except for monotony indices (internal and external load) that G2 showed greater values than G1 and G3. Coaches and practitioners should plan the training “doses” to reduce disparities of these different match’s participation and congested schedule weeks.

## Introduction

In professional soccer, teams should compete in up to 50 matches per season^1^. In addition, the elite teams typically compete domestic leagues and national championships resulting in periods of congested fixtures. The accumulated matches over a short period potentially result in residual fatigue and underperformance due to insufficient time for physical and cognitive recovery^2^. However, no differences have been reported in match running performance (e.g., total distance covered at different speed thresholds) both in short and long periods of match congested fixtures^3–6^. This stable match running performance could imply that elite players can maintain the physical performance across congested fixture periods^1^. An alternative explanation can be related to the coach’s planned strategies of player rotation during these critical periods. In contrast, the congested schedule potentially decreases muscle stiffness^7^, and increases the physiological stress, muscle damage^8^, and strength deficits^9^.

However, few studies have explored the training load (TL) accumulated during match congested fixtures^10^. TL can be described as internal (i.e., psychophysiological response) and external (i.e., quality and quantity of exercise), which will ultimately determine the design of training programs^11^. Usually, the few studies that have investigated the effects of the congested fixture on TL used distance-based metrics of external load (e.g., total distance covered at different speed thresholds^12–14^ or session-rating of perceived exertion (s-RPE)^10^. The sRPE is considered a valid indicator of internal load in soccer training and match-play^15^. However, to the best of our knowledge, no previous studies considered jointly external (distance- and accelerometry-based measures) and internal TL (session-RPE) to verify the potential effects of the congested fixture on load accumulation in professional soccer. Also, the derived indices obtained from TL calculation, such as training monotony and training strain, should be described aiming to quantify load variations within the analyzed week^16^.

In addition, the TL is typically planned to ensure readiness to play in the ensuing official match; however, usually, the rules only allow the participation of, at most, 14 players (before the Covid-19 pandemic) or 16 players (during the Convid-19 pandemic)^17^. Previous studies reported greater weekly TL for the starters than for the non-starters^12^. Coaches and practitioners should organize compensatory strategies for players who did not participate in the match or who played few minutes (e.g., < 60 min) to compensate for the missing daily load^18^. Therefore, a well-implemented monitoring cycle (external and internal TL), considering independent variables as congested fixtures and level of participation during the matches, can provide valuable information to coaches and sports scientists to optimize players’ recovery and performance during the season.

Therefore, this study aimed to verify the effects of congested fixtures and matches’ participation on internal and external workload indices in professional Brazilian soccer players. We expected that congested weeks presented greater values of internal and external workload indices than regular weeks. Also, we expected that players who played ≥ 60 min presented greater values of external and internal workload indices compared to players who played < 60 min and the players who did not participate of the match.

## Methods

### Study design

Professional soccer players were monitored during 119 training sessions and 33 matches over 21 weeks during the 2019 competitive season of the Brazilian National 2nd Division League in one reference team. Weeks were classified as congested (two or more matches within a 7-day period) and regular (one match or less within a 7-day period) based on match exposure^2, 19^. Thus, we analyzed 11 congested (n = 1346 individual observations) and 10 regular weeks (n = 1465 individual observations). The players were divided based on the matches’ participation^19, 20^: (1) players who played ≥ 60 min (G1; n = 1034 individual observations); (2) players who played < 60 min (G2; n = 344 individual observations); and (3) players who did not participate of the match (G3; n = 1435 individual observations). The players were classified according to five positional roles: (1) central defenders (n = 482 individual observations); (2) external defenders (n = 555 individual observations); (3) central midfielders (n = 623 individual observations); (4) external midfielders (n = 744 individual observations); (5) forwards (n = 407 individual observations). The playing positions were set as covariates. The acute load (weekly sum of load—Eq. 1), training monotony (mean of training load during the seven days of the week divided by the standard deviation of the training load of the week—Eq. 2), and training strain (multiplication of acute load by the training monotony—Eq. 3) were calculated by each internal and external load measures^16^.

1 $$Acute\, Load = weekly\, sum\, of\, load\, (monday + tuesday + wednesday + thursday + friday + saturday + sunday)$$

2 $$Training\, monotony = ((Acute\, Load)/7)/(\sigma Acute\, Load))$$

3 $$Training \,Strain = Acute\, Load * Training \,monotony$$

Note: $\sigma$ = standard deviation.

### Participants

Twenty-nine male outfield professional soccer players were monitored daily (age 26 ± 4 years; height 179 ± 7 cm; body mass 78 ± 8 kg; professional experience 6 ± 4 years; n = 5 central defenders; n = 6 external defenders; n = 6 central midfielders; n = 8 external midfielders; n = 4 forwards). The study was approved by the local Human Research Ethics Committee (School of Physical Education and Sport of Ribeirão Preto, University of São Paulo; protocol no. 61884716.9.0000.5659).

### Internal load monitoring

To minimize errors and increase the accuracy of the answers, the Borgs' scale (CR10) was previously presented to the team for familiarization on daily basis for two weeks. Approximately thirty minutes after the end of training sessions or matches, players were asked to answer "*How was your workout*?". Internal load (rating of perceived exertion-based training load (sRPE)), reported as arbitrary units (AU), was calculated by multiplying the adapted version of Borg’s CR10 score^21^ by training or match duration^16^. Therefore, the following internal load indicators were calculated^16^: acute-sRPE, monotony-sRPE, and strain-sRPE.

### External load monitoring

Each player wore a 10-Hz global position system (GPS) integrated with a 400-Hz Tri-Axial Accelerometer and 10-Hz Tri-Axial magnetometer (Playertek, Catapult Innovations, Australia) in order to record distance- and accelerometry-based measures during the training sessions and matches. The devices were fitted in the upper back of the players using adjustable harnesses and were activated 15 min before the data collection, in accordance with the manufacturer’s instructions. During all data collection period the players used the same GPS device and the metrics obtained for each training or match were: (1) total distance covered (TD, meters); (2) total distance covered in high-intensity running (HIR, ≥ 18 km·h^−1^, meters); (3) total distance covered in high-acceleration (Acc, ≥ 2 m·s^−2^, m); (4) total distance covered in high-deceleration (Dec, ≤  − 2 m·s^−2^, m). The speed and accelerometry thresholds used are similar to those reported in previous studies^19, 22, 23^. Therefore, the following external load indicators were calculated^16^: acute-TD, monotony-TD, strain-TD; acute-HIR, monotony-HIR, strain-HIR; acute-Acc, monotony-Acc, strain-Acc; Acute-Dec, Monotony-Dec, Strain-Dec.

### Statistical analysis

The Kolmogorov–Smirnov and Levene tests were used to check the normality of the data distribution and homogeneity of variance, respectively. Data are presented as mean and standard deviation (SD). A multivariate general linear model was used to verify the independent and interactive effects of the fixed factors (i.e., match schedule, congested vs. regular weeks; matches’ participation, G1 vs. G2 vs. G3) on the internal and external measures (dependent variables). The Bonferroni test was used to verify the pairwise differences. The level of significance was pre-fixed in 5% (*p* < 0.05). The playing positions (i.e., central defenders, external defenders, central midfielders, external midfielders, forwards) were computed as a covariate. The magnitude of differences was tested using the standardized effect size (ES) of Cohen (d), following the thresholds^24^: 0.0–0.20, small; 0.21–0.50, medium; 0.51–0.80, large, > 0.8 very large. The partial eta squared (η^2^) was classified as: 0.00–0.059 (small), 0.06–0.14 (medium), and ≥ 0.15 (large)^24^. All statistical analyses were run using the SPSS software (version 25.0; IBM SPSS Inc., Chicago, IL, USA).

### Ethical approval

The training coaches of the club, after obtaining permission from the relevant authorities and the head coach of the club, conducted this research. Before commencing the study, it also received the approval of the research ethics committee from the University of São Paulo (61884716.9.0000.5659). All players were informed of the purpose of the study before completing the informed consent. All stages of this study were carried out based on the ethical principles in the Helsinki Declaration.

## Results

Table 1 shows the effects of congested fixture and matches’ participation on acute TL, monotony, and strain of the weekly internal load. The multivariate general linear model showed significant effect on match schedule (F = 10,433˗101,428; *p* < 0.001˗0.001; η2 = 0.021˗0.170, small˗large). The post-hoc test showed that G1 (*p* < 0.001, ES = 0.46 − 1.09, medium − very large), G2 (*p* < 0.001, ES = 0.85 − 1.50, large − very large) and G3 (*p* < 0.001, ES = 0.71 − 1.56, large − very large) presented greater values of acute-sRPE and strain-RPE in regular compared to congested weeks. In addition, G1 showed higher values of monotony-sRPE in regular than congested weeks (*p* < 0.001, ES = 1.26, very large).

**Table 1:** Mean (standard deviation) of the acute, monotony, and strain indices for the rate-of-perceived-exertion-based training load (sRPE) according to congested fixture and matches’ participation.

Variables	Regular weeks			Congested weeks		
	G1	G2	G3	G1	G2	G3
Acute-sRPE (A.U.)	2417.7 (640.7)*^,#,Ø^	1743.6 (500.9)**	1654.4 (445.2)***	2133.8 (588.4)^#,Ø^	1038.7 (434.4)	929.3 (484.7)
Monotony-sRPE (A.U.)	1.3 (0.4)*	1.7 (0.7)^*¶*^	1.4 (0.6)	0.9 (0.2)	1.6 (0.7)^*¶*^	1.3 (0.8)^β^
Strain-sRPE (A.U.)	3174.3 (1503.4)*^,Ø^	2970.8 (1555.9)**	2484.3 (1406.6)***	1869.7 (766.1)^Ø^	1782.3 (1234.6)	1422.7 (1564.6)

G1 = players who played ≥ 60 min; G2 = players who played < 60 min; G3 = players who did not participate of the match. * G1: Regular week > Congested week; ** G2: Regular week > Congested week; *** G3: Regular week > Congested week; ^#^ G1 > G2; ^Ø^ G1 > G3; ^*¶*^ G2 > G1; ^β^ G3 > G1.

The multivariate general linear model showed significant effect on matches’ participation (F = 9826–189,455; *p* < 0.001; η2 = 0.038˗0.434, small˗large). The post-hoc test showed that G1 presented greater values of acute-sRPE than G2 (*p* < 0.001, ES = 1.17–2.11, very large) and G3 (*p* < 0.001, ES = 1.38–2.23, very large) in regular and congested weeks. Furthermore, G1 showed superior values of strain-sRPE than G3 (*p* = 0.001, ES = 0.36–0.48, medium) in regular and congested weeks. However, reduced values of monotony-sRPE were verified in G1 compared to G2 (*p* < 0.001, ES = 1.36, very large) and G3 (*p* < 0.001, ES = 0.68, large) in congested weeks, while G2 presented greater values than G1 in regular weeks (*p* = 0.005, ES = 0.70, large).

Table 2 presents the effects of congested fixture and matches’ participation on acute, monotony, and strain distance-based measures (TD, HIR). The multivariate general linear model showed significant effect on match schedule (F = 57,396˗180,273; *p* < 0.001; η2 = 0.104˗0.267, medium˗large). The post-hoc test showed that G1 (*p* < 0.001, ES = 1.08–1.48, very large), G2 (*p* < 0.001, ES = 0.49–1.71, medium − very large) and G3 (*p* < 0.001, ES = 1.10–1.54, very large) showed greater values of acute-TD, monotony-TD, and strain-TD in regular compared to congested weeks. In addition, regular weeks presented higher values of acute-HIR and strain-HIR for G1 (*p* =  < 0.001–0.007, ES = 0.32–0.84, medium − very large), G2 (*p* < 0.001, ES = 0.86 − 1.24, very large) and G3 (*p* < 0.001, ES = 1.01 − 1.12, very large) than congested weeks. Also, G1 (*p* < 0.001, ES = 1.56, very large) and G3 (*p* < 0.001, ES = 0.94, very large) demonstrated greater values of monotony-HIR in regular versus congested weeks.

**Table 2:** Mean (standard deviation) of the acute, monotony, and strain indices for the total distance covered (TD) and total distance covered in high-intensity running (HSR; ≥ 18 km·h^−1^) according to congested fixture and matches’ participation.

Variables	Regular weeks			Congested weeks		
	G1	G2	G3	G1	G2	G3
Acute-TD (m)	28,086.8 (5021.0)*^,#,Ø^	23,180.7 (5469.2)**^,§^	20,185.2 (5230.3)***	22,373.2 (5582.5)^#,Ø^	13,967.3 (5316.9)	12,063.1 (5333.1)
Monotony-TD (A.U.)	1.7 (0.6)*	2.3 (1.1)**^,*¶*,§^	1.5 (0.5)***	1.0 (0.3)	1.8 (0.9)^*¶*,§^	1.0 (0.4)
Strain-TD (A.U.)	49,273.8 (23,344.2)*^,Ø^	55,478.8 (31,358.2)**^,§^	32,162.2 (16,086.7)***	22,676.3 (9932.9)^Ø^	27,011.3 (19,720.3)^§^	13,206.0 (10,917.9)
Acute-HIR (m)	2088.8 (735.8)*^,#,Ø^	1889.3 (689.2)**^,§^	1207.5 (517.2)***	1847.5 (779.5)^#,Ø^	1139.5 (511.4)^§^	719.9 (441.3)
Monotony-HIR (A.U.)	1.1 (0.3)*	1.5 (0.4)^*¶*,§^	1.1 (0.4)***	0.7 (0.2)	1.3 (0.6)^*¶*,§^	0.8 (0.2)
Strain-HIR (A.U.)	2362.9 (1302.3)*^,Ø^	2819.9 (1427.7)**^,§^	1431.5 (837.5)***	1433.5 (857.5)^Ø^	1636.2 (1307.0)^§^	619.7 (594.8)

G1 = players who played ≥ 60 min; G2 = players who played < 60 min; G3 = players who did not participate of the match. * G1: Regular week > Congested week; ** G2: Regular week > Congested week; *** G3: Regular week > Congested week; ^#^ G1 > G2; ^Ø^ G1 > G3; ^*¶*^ G2 > G1; ^§^ G2 > G3.

The multivariate general linear model showed significant effect of matches’ participation (F = 44,105˗150,903; *p* < 0.001; η2 = 0.152˗0.379, large). The post-hoc test showed that G1 presented greater values of acute-TD than G2 (*p* < 0.001, ES = 0.93 − 1.54, very large) and G3 (*p* < 0.001, ES = 1.54 − 1.89, very large) in regular and congested weeks. G2 also presented higher values of acute-TD (*p* = 0.001, ES = 0.56, large) and strain-TD (*p* = 0.004, ES = 0.94, very large) than G3 in regular weeks. Regarding monotony-TD/HIR, G2 showed greater values than G1 (*p* < 0.001, ES = 0.58 − 1.34, large − very large) and G3 (*p* < 0.001, ES = 0.58 − 1.12, large − very large) in regular and congested weeks. G1 showed higher values of acute-HIR than G2 (*p* < 0.001, ES = 1.07, very large) and G3 (*p* < 0.001, ES = 1.78, very large) in congested weeks, while G1 showed greater values than G3 in regular weeks (*p* < 0.001, ES = 1.39, very large). The strain-HIR was superior in G1 and G2 than G3 in regular and congested weeks (*p* < 0.001, ES = 0.85 − 1.19, very large).

The effects of congested fixture and matches’ participation on accelerometry-based measures (Acc and Dec) are demonstrated in Table 3. The multivariate general linear model showed significant effect on match schedule (F = 100,479˗181,920; *p* < 0.001; η2 = 0.169˗0.269, large). The post-hoc test showed that G1 (*p* < 0.001, ES = 1.08 − 1.37, very large), G2 (*p* < 0.001 − 0.001, ES = 0.58 − 1.80, large − very large) and G3 (*p* < 0.001, ES = 1.11 − 1.38, very large) showed higher values of acute-Acc, acute-Dec, monotony-Acc, monotony-Dec, strain-Acc, and strain-Dec in regular compared to congested weeks.

**Table 3:** Mean (standard deviation) of the acute, monotony, and strain indices for the total distance covered in high-acceleration (Acc; ≥ 2 m·s^-2^) and high-deceleration (Dec; ≤ -2 m·s^-2^) according to congested fixture and matches’ participation.

Variables	Regular weeks			Congested weeks		
	G1	G2	G3	G1	G2	G3
Acute-Acc (m)	888.9 (206.7)*^,Ø^	852.7 (213.5)**^,§^	707.4 (206.6)***	650.2 (192.9) ^#,Ø^	500.6 (197.4)	432.3 (192.9)
Monotony-Acc (A.U.)	2.0 (1.1)*^,Ø^	2.1 (1.0)**^,§^	1.5 (0.6)***	1.0 (0.3)	1.6 (0.7)^*¶,*§^	0.9 (0.3)
Strain-Acc (A.U.)	1834.1 (1225.0)*^,Ø^	1877.9 (1052.2)**^,§^	1134.8 (667.0)***	675.0 (393.4)^Ø^	856.7 (606.9)^§^	443.2 (350.1)
Acute-Dec (m)	1079.6 (255.2)*^,Ø^	1012.5 (240.9)**^,§^	831.0 (258.2)***	811.2 (241.5)^#,Ø^	578.6 (240.3)	493.4 (229.1)
Monotony-Dec (A.U.)	1.7 (0.8)*	2.1 (0.9)**^,*¶,*§^	1.5 (0.7)***	0.9 (0.2)	1.5 (0.7)^*¶,*§^	0.9 (0.3)
Strain-Dec (A.U.)	1921.6 (1144.1)*^,Ø^	2194.3 (1231.9)**^,§^	1330.4 (852.6)***	768.6 (378.6)^Ø^	982.1 (796.4)^§^	489.7 (395.6)

G1 = players who played ≥ 60 min; G2 = players who played < 60 min; G3 = players who did not participate of the match. * G1: Regular week > Congested week; ** G2: Regular week > Congested week; *** G3: Regular week > Congested week; ^#^ G1 > G2; ^Ø^ G1 > G3; ^*¶*^ G2 > G1; ^§^ G2 > G3.

The multivariate general linear model showed significant effect on matches’ participation (F = 25,786˗79,009; *p* < 0.001; η2 = 0.095˗0.242, medium˗large). The post-hoc test showed that G1 presented greater values of acute-Acc/Dec than G2 (*p* < 0.001, ES = 0.77 − 0.96, large − very large) and G3 (*p* = 0.001, ES = 1.13 − 1.35, very large) in congested weeks, while G1 and G2 was higher than G3 for acute-Acc/Dec and strain-Acc/Dec in regular weeks (*p* < 0.01, ES = 0.59 − 0.96, large − very large). In congested weeks, the monotony-Acc/Dec were superior in G2 compared to G1 (*p* < 0.001, ES = 1.11 − 1.16, very large) and G3 (*p* < 0.001, ES = 1.11 − 1.30, very large). In regular weeks, G1 and G2 were superior to G3 for monotony-Acc (*p* < 0.001, ES = 0.56 − 0.73, large) and G2 was superior to G1 and G3 for Monotony-Dec (*p* < 0.001, ES = 0.47 − 0.74, medium − large). The strain-Acc/Dec presented greater values for G2 compared to G3 in congested weeks (*p* = 0.04, ES = 0.78 − 0.83, large − very large). G1 also presented superior values of Strain-Dec than G3 in congested weeks (*p* = 0.02, ES = 0.72, large).

## Discussion

This study investigated the effects of congested fixtures and matches’ participation on internal and external load in professional soccer. Our initial hypothesis was partially confirmed. First, we expected that congested weeks presented greater values of internal and external workload indices than regular weeks. However, in general, the findings showed that independent of the levels of participation during the matches, regular weeks presented greater acute TL, monotony, and strain indices for internal and external workload measures than congested weeks. Second, we expected that players who played ≥ 60 min (G1) presented the greater values of external and internal workload indices compared to players who played < 60 min (G2) and the players who did not participate of the match (G3). We verified that G1 presented the highest values for most of the workload indices in both regular and congested weeks, except for monotony indices (internal and external load), and that G2 showed greater values in these indices than G1 and G3, independent of the week being congested or regular.

Previous studies showed no significant effects of the congested fixture on match running performance compared to regular weeks with fewer matches^1, 25^. However, these studies did not report measures the TL during these weeks (regular vs. congested). Our study showed that independent of the level of participation during the matches, regular weeks presented greater internal and external TL than congested weeks. These findings can explain, at least in part, the absence of differences of running outputs during the matches. In addition, Gualtieri, Rampinini^14^ reported that starters obtained higher internal and external workload compared to non-starters during congested weeks. Here, the players who played ≥ 60 min (G1) had higher internal and external load values than players who played < 60 min (G2) and those who only trained (G3) during regular and congested weeks. Despite having methodological differences, our study corroborates that starters are exposed to higher levels of loads throughout the week. However, in general, we observed that G2 presented higher monotony indices than G1 and G3. These results demonstrated the importance of load monitoring considering the different levels of participation during the matches in regular and congested weeks. The coaches and practitioners should reduce the disparities of the internal and external loads to these different match’s participation and congested schedule weeks, avoiding load “spikes” and under or overload.

A previous study has shown that professional Brazilian soccer players covered ~ 10,000 m during a regular match (18% of this distance in high-intensity actions: > 16.1 km·h^−1^)^26^. In the present study, comparing the groups that played (G1 and G2) with the group that did not play (G3) it is possible to notice that the differences for the analyzed variables refer only to the inclusion of the match load. For example, in regular weeks, G1 presented higher values than G3 for the internal load (acute s-RPE: 2417.7 A.U. vs. 1654.4 A.U.) and for the external load (e.g. acute-TD: 28,086.8 m vs. 20,185.2 m). These findings were also observed in congested weeks (G1 > G3).

The training monotony and strain derived from the external load have been investigated in soccer players ^12^. Here, the players who played < 60 min (G2) presented the highest monotony indexes for internal and external loads. Expressive values of training monotony indicate low weekly load variation. In addition, we observed lower TL variation (i.e. high monotony index) for G2 during regular compared to congested weeks (e.g., monotony-DEC in regular week: 2.1 A.U. vs. monotony-DEC in congested week: 1.5 A.U.). In this study, one and two days after the match (i.e. Match + 1 or Match + 2), the coaches applied recovery strategies to players who played ≥ 60 min (G1). In contrast, in these days mainly after the away matches, the coaches were unable to compensate for the missing match load for the players who played < 60 min (G2), mainly due to the travel and/or the logistic of the training (e.g. space, number of players available to train). Also, during the following days of the week (e.g. Match + 3 and Match + 4), these players (G2) did not receive high values of TL because, usually, they were selected for the next match.

Our study has some limitations. First, we did not stratify the weekly loads for each day of the week, which could help better understand the distribution of the internal and external loads. Second, we analyzed only one team during the season. More studies should include other teams and divisions to generalize the results found. Finally, we did not consider contextual factors in our analyses (e.g., situational variables). The inclusion of these factors could help to elucidate the differences found and the behavior of loads in a more holistic way^19^.

## Conclusions

Our findings help to identify the profile of internal and external loads during regular and congested weeks of the whole in-season period of professional soccer players. We found that distance- and accelerometry-based measures and sRPE presented higher values during regular vs. congested weeks. In addition, the players who played ≥ 60 min presented the highest values of acute weekly load. In addition, the players who played < 60 min presented the highest values of monotony indexes both for internal and external loads. The coaches and practitioners should make efforts to reduce the disparities of the workloads to these different levels of participation during the matches and regular/congested weeks, avoiding load “spikes” and under or overload. Coaches should organize friendly games or large-sided games (≥ 7-a-side; > 250 m^2^ per player) as a compensatory training strategy for players who did not participate in the match or who played < 60 min to compensate for the missing match load. Therefore, we recommend that coaches could create specific training strategies to vary the TL for these players (G2) in regular weeks, especially two, three, and four days after the matches (e.g., using high-intensity interval training: running in a straight line or change-of-direction; muscular strength; small-sided games).

## Supplementary Information

Supplementary Information.

## Data Availability

The datasets generated during and analyzed during the current study are available from the corresponding author on reasonable request.
